# New GO-based measures in multiple network alignment

**DOI:** 10.1093/bioinformatics/btae476

**Published:** 2024-07-31

**Authors:** Kimia Yazdani, Reza Mousapour, Wayne B Hayes

**Affiliations:** Department of Computer Science, University of California, Irvine, CA 92697-3435, United States; Department of Computer Engineering, Sharif University of Technology, Tehran 1458889694, Iran; Department of Computer Science, University of California, Irvine, CA 92697-3435, United States

## Abstract

**Motivation:**

Protein–protein interaction (PPI) networks provide valuable insights into the function of biological systems. Aligning multiple PPI networks may expose relationships beyond those observable by pairwise comparisons. However, assessing the biological quality of multiple network alignments is a challenging problem.

**Results:**

We propose two new measures to evaluate the quality of multiple network alignments using functional information from Gene Ontology (GO) terms. When aligning multiple real PPI networks across species, we observe that both measures are highly correlated with objective quality indicators, such as common orthologs. Additionally, our measures strongly correlate with an alignment’s ability to predict novel GO annotations, which is a unique advantage over existing GO-based measures.

**Availability and implementation:**

The scripts and the links to the raw and alignment data can be accessed at https://github.com/kimiayazdani/GO_Measures.git

## 1 Introduction

### 1.1 Motivation

Proteins perform their role in the biological system by physically interacting with each other in the cell. A protein–protein interaction (PPI) network highlights the functions of the proteins in the cell by representing the interactions between its proteins. Gene Ontology (GO) provides biological annotations for proteins across different species (Gene Ontology Consortium 2008). Aligning multiple PPI networks of multiple species can allow us to discover functions of human proteins by using the information of the aligned proteins in other networks.

The shape of a protein determines its surface and as a result, determines the set of other proteins that it can physically interact with. Two proteins with no sequence similarity can have a close similarity in these surfaces and have a similar function as well (Furuse *et al.* 1998, Schlicker *et al.* 2006). Additionally, two proteins with very close sequences can have a completely different shape structure and completely different functions (Kabsch and Sander 1984, Morrone *et al.* 2011). Moreover, sometimes a small change in the sequence can result in a completely different set of interaction partners and therefore a completely different functionality (Kimchi-Sarfaty *et al.* 2007; Zhao *et al.* 2014). Thus, while sequence is clearly correlated with protein function, the relation is neither direct nor immutable. Since physical PPI interactions can be directly measured, these can provide a more direct path than sequence to inferring functions implied by network topology (Brückner *et al.* 2009). Therefore, PPI networks directly represent the interactions and they can be used to predict the functionality of proteins in a way that sequence similarity cannot (Wang *et al.* 2022a). Additionally, the PPI networks of different species are deeply connected to each other. For instance, 80% of human genes have a direct ortholog in mouse genes (Pennacchio 2003). As proteins are the products of genes, we can use network alignments to discover regions of similar PPI network topology between different species and transfer functional information between the protein with more available functional information to one that is less studied.

However, these networks are often noisy and incomplete (Kuchaiev *et al.* 2009), which can limit their utility (Wang *et al.* 2022b). Multiple network alignment may help boost the signal-to-noise ratio by allowing the correlation between more than two networks (Wang *et al.* 2022a). Given that network alignment is a hard problem (NP-complete) even in the absence of noise, the fact that they are significantly noisy makes the problem even more difficult. Therefore, it is important to assess the quality of all solutions in a careful and biologically relevant way. There are many sources of information available that could be used to guide a network alignment, including graph-theoretic topology measuring common interactions between the networks, sequence similarity of aligned proteins, and functional similarity as described by GO terms or KEGG pathways. Most network alignments use one or both of the first two (Kalaev *et al.* 2008, Trung *et al.* 2020).

Clearly, any information used to guide an alignment cannot be used to judge its quality after-the-fact, as that would induce circular reasoning. Instead, we can evaluate the alignment using measures independent of those used to guide its construction. It has been shown that common topology relates closely to common function (Pržulj *et al.* 2004, Milenković and Pržulj 2008, Davis *et al.* 2015, Wang *et al.* 2022a). As a result, in biological network alignment, it is crucial to consider the functional information exposed by topologically driven network alignments. Functional information can be garnered from many sources, including *post hoc* sequence analysis, analysis of the fraction of common orthologs aligned, as well as direct functional information encoded in databases such as KEGG, CORUM, and the GO (Ashburner *et al.* 2000, Kanehisa and Goto 2000, Ruepp *et al.* 2008). Here, we focus on the latter.

### 1.2 Gene ontology

The Gene Ontology Consortium (2008) has a hierarchical structure and includes a large number of descriptive terms to describe BP, Cellular Components (CC), and Molecular Functions (MF) that occur in a cell. Some proteins are highly studied and are annotated by many GO terms while others are less studied and have few or no GO annotations. Near the top of the GO term hierarchy, the terms are very general and many proteins can be annotated by those terms. Lower in the hierarchy, the terms are more specific and annotate fewer proteins.

Like PPI networks, GO annotations are noisy and incomplete—an issue we must keep in mind though we do not directly address it in this article. However, even disregarding this noise, there are many possible ways to use GO annotations to measure the quality of a multiple network alignment. To our knowledge, all existing measures such as Semantic Similarity of Proteins work on an isolated set of proteins without regard to a wider context, such as how (Kazemi and Grossglauser 2020) evaluated each cluster of aligned proteins independently. There is evidence suggesting that the existing GO-based measures in pairwise network alignment do not have predictive value. In the context of multiple network alignment, existing methods measure the GO-based functional similarity within a *cluster* of aligned proteins (Hayes 2024). In these measures, the final score of the measure is the mean of the score for across all clusters. However, an alignment is more than the isolated sets of its aligned proteins. The alignment itself is built in a global way and the aligned sets are dependent on each other. In an alignment, each cluster of aligned proteins might have a shared GO term that is very specific and therefore would receive a high score based on its information content by protein semantic similarity. The same GO term might be aligned randomly in the rest of the alignment but since we are looking at each cluster of proteins in an isolated way, we would score the alignment high. On the other hand, assume we have a network that does not share GO terms with the other networks. The best possible alignment that we can create would still receive a low score from protein semantic similarity since the clusters cannot have high information content.

Additionally, there are two measures, GO correctness (GC) (Vijayan and Milenković 2018) and GO specificity (GS) (Gligorijević *et al.* 2016), that only analyze whether each cluster of aligned proteins have at least one common GO term or not, and then score the alignment based on the fraction of those aligned proteins. The difference between the two measures is that GC first breaks down the cluster of aligned proteins to aligned protein pairs and then calculates the same equation on the resulting pairs. One problem with this approach is that it does not distinguish between sharing a very common GO term—which can happen merely by chance—versus sharing a highly specific GO term, which is significant. If we have a random alignment and a common GO term, it will be shared in many clusters and receive a very high score. As confirmed by the results of this article, this leads to early saturation of these measures. The only provided solution to this is to use an arbitrary cutoff to consider only the more specific GO terms in scoring. The problem with this approach is that the GO terms at the same depth do not necessarily have the same specificity. Sometimes, a GO term at a higher level might be even more specific (Resnik 1999). There is no clear criteria by which one can choose the arbitrary cutoff. These measures are also not global in the sense that they consider GO terms only within each cluster of aligned protein with no regard to its context across the whole alignment. In contrast, our measure uses the frequency of a GO term across the entire PPI network of one species as a direct measure of specificity, and weights it as such without any cutoffs.

For demonstrating the functional relevance of the measures, we used both BioGRID networks and IID networks (Wang *et al.* 2022a). The BioGRID database (Chatr-Aryamontri *et al.* 2017) is an extensive resource for PPI networks which is released monthly and thus constantly updated and therefore we have used it as a part of our validation. However, due to the skewed sample derived from the literature for different species (Gillis *et al.* 2014), we have also used IID for another part of our validation. The IID networks comprise PPI networks for 11 mammalian species, where the networks with fewer documented interactions have been augmented with edges based on known common interactions between the nodes in different species, such as interologs. Thus, even though they are partly synthetic, the IID networks are an ideal test-bed for multiple network alignment due to their having high topological similarity by construction—a construction with plausible biological backing.

Our measures provide a practical framework for evaluating the quality of multiple network alignments, which has potential application in helping researchers to identify the most biologically relevant aligned regions. Our work addresses the above challenges by proposing two function-based quality measures for assessing the biological quality of multiple network alignment based on a single GO term. The information from multiple GO terms can then be combined in a second step using mean. Additionally, through extensive experiments on both synthetic and real biological networks, we demonstrate that Squared G score (SGS) and Exposed G Score (EGS) are highly correlated with the number of recovered orthologs, an objective evidence of alignment quality. We also demonstrate that our measures can work with different filters such as using thresholds on frequency or GO terms of specific category of GO terms such as exclusively including BP GO terms. Moreover, we show that our proposed measures can improve the precision of cross-species GO term prediction, highlighting their functional relevance.

## 2 Materials and methods

### 2.1 Definitions

We define cluster as a group of proteins that are aligned together across different networks. We look at multiple network alignment in a 1–1 context which means every node is aligned to exactly one cluster, and a cluster can contain at most one node from each network. While this is a slight simplification from biological reality (e.g. it neglects *paralogs*) two proteins from the same network that are nearly identical—it is a decent approximation and a good starting point. Additionally, from each network, at most one node is allowed in a cluster. We refer to a single GO term denoted by *g* and use the term *g*-annotated for the nodes that are annotated with GO term *g* and the term un-annotated for the nodes that are not annotated with *g*. Later, we combine the scores for all of the GO terms.

To provide a visual analogy of multiple network alignment, we schematically view an alignment as a set of “levels,” with each network occupying one level, with its proteins arranged on a grid with each gridpoint occupied by at most one protein from each network. A cluster of aligned proteins consists the set of proteins—at most one from each network—occupying the same gridpoint at different levels; we call such a cluster a “tower” of proteins. Given tower *T*, we use *a_T_* for the number of proteins in *T* that are annotated by *g*. For *k* undirected graphs $G_{i},i=1,\ldots ,k$, let *G_i_* have *n_i_* nodes, *λ_i_* of which are annotated with *g*. We also use *n*_0_ to indicate the number of grids that we can match proteins to, which can be equal or greater than the size of the biggest network. Without loss of generality, we assume two conventions for sorting the networks: **node-sorted**, where the networks are sorted by number of nodes: $n_{1}\geq n_{2}\geq \ldots \geq n_{k}$; and **lambda-sorted**, where $\lambda_{1}\geq \lambda_{2}\geq ,\ldots \geq \lambda_{k}$.

### 2.2 Equations

#### 2.2.1 Preliminaries

Recall that, for now, we are working with a single GO term *g*. Thus, each protein in a cluster (tower) is either annotated by *g*, or not.

We introduce two measures below that are used after-the-fact, never to guide an alignment. Since the goal of network alignment is to cluster together proteins with similar function, we consider an alignment that “concentrates” more *g*-annotations into fewer towers is better than spreading *g*-annotations around many towers. We propose two ways to measure this concentration: we can either reward towers that have more *g*-annotations than average (the SGS score below), or we can reward the *entire alignment* if all the *g*-annotations are concentrated into fewer towers [the Exposed G (EG) value below]. Thus, better alignments have a larger SGS score, but a *smaller* EG value. EG is subtracted from the maximum EG value in the numerator, so the EGS is also higher if we have a better alignment.

We have normalized both of these measures in a way that they output exactly zero for the worst-case multiple network alignment between a certain set of networks, and exactly 1 for the best-case.

#### 2.2.2 Squared G score

**Figure 1.**
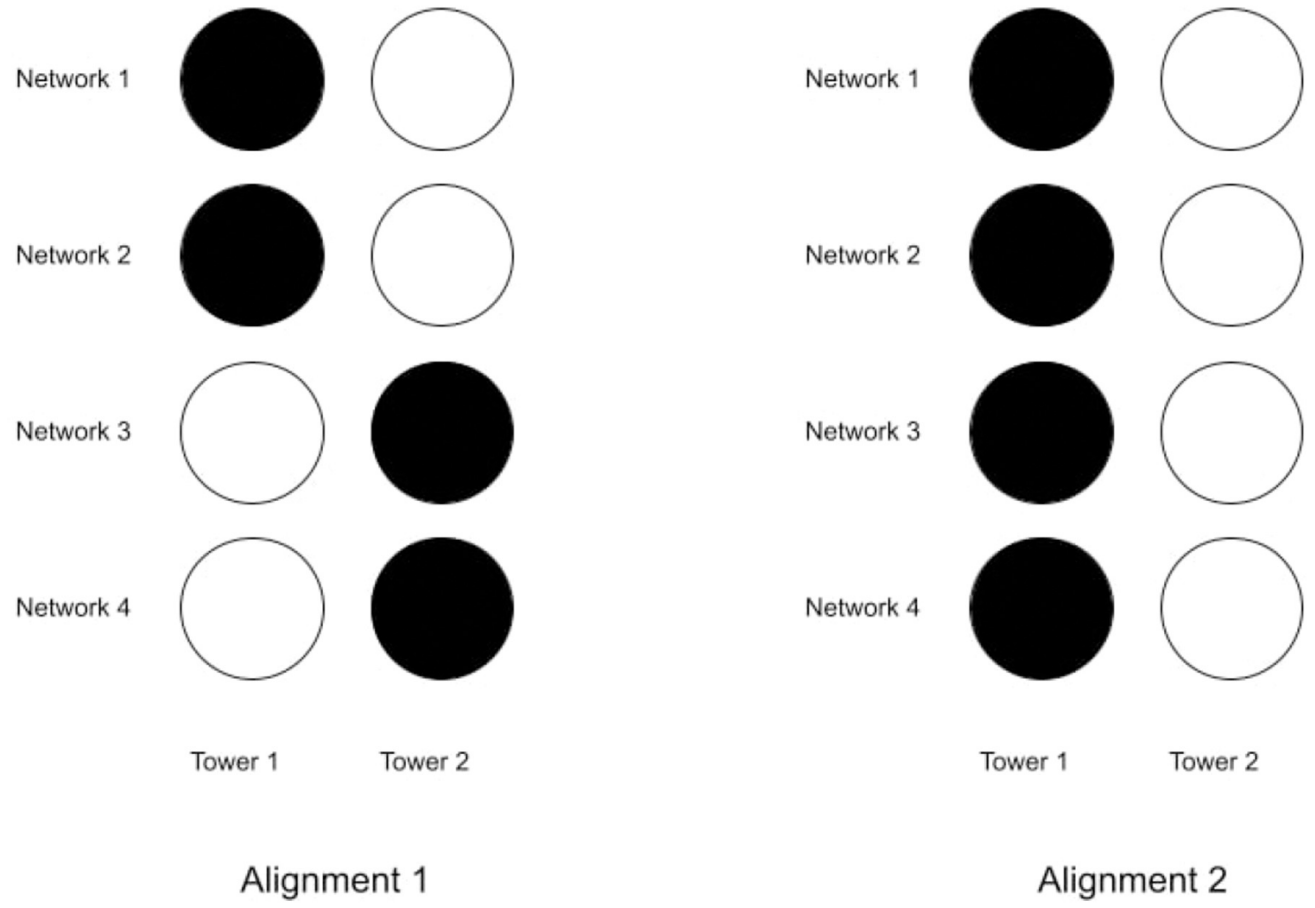
The figures show two different alignments. The black circles represent the *g*-annotated nodes and the white circles represent unannotated nodes. Each row represents a network and each column represents a tower or a cluster of aligned nodes. Alignment 2 has concentrated all *g*-annotated nodes in the same tower which means it is a better alignment than Alignment 1

We assume the networks are lambda-sorted for this section. This measure computes the sum of the squared number of *g*-annotated nodes in each tower. Another simpler approach that might come to mind is to just add the number of aligned *g*-annotated nodes without squaring them. However, the weakness of this approach is demonstrated in Fig. 1. Using this approach would give a score of 4 to both alignments whereas Alignment 2 is a better one. Using SGS would give a score of $2^{2}+2^{2}=8$ to Alignment 1 and $4^{2}=16$ to Alignment 2. Therefore, SGS offers a better differentiation for the quality of alignments.

An alignment *F* is the set of *k* 1-to-1 mappings from the nodes of each network to the gridpoint. We define SGS for tower *T* as $|a_{T}{|}^{2}$, the square of the number of annotated proteins. Then, the global score of *F* is

(1) $$\text{SG}(F)=\sum_{T}|a_{T}{|}^{2}.$$

To normalize the SGS score between 0 and 1, we use its minimum and maximum possible value, which is computed as follows:

(2) $$\text{Min}(\text{SG})=\sum_{i=1}^{k}\lambda_{i}$$

(3) $$\text{Max}(\text{SG})=k^{2}\lambda_{k}+\Sigma_{i=1}^{k-1}i^{2}(\lambda_{i}-\lambda_{i+1})$$

(4) $$\text{SGS}=\frac{\text{SG}-\text{Min}(\text{SG})}{\text{Max}(\text{SG})-\text{Min}(\text{SG})}$$

where *k* is the number of networks in the alignment. It is important to note that by subtracting the minimum possible value for SG, we are giving no score to the towers that only have one *g*-annotated node. We can obtain a lower bound for a situation where all the GO terms remain unaligned by adding all the *λ_i_* values for different networks. Conversely, we can calculate an upper bound for the maximum value for SG by maximizing the number of aligned *g*-annotated nodes in the towers. To do this, we first align all the GO terms in the network with the smallest lambda (*λ_k_*) with *λ_k_ g*-annotated nodes from all the other *k—*1 networks. Then we match the remaining *g*-annotated nodes in the network with the second smallest *λ_i_* with the same number of *g*-annotated nodes in the *k—*2 other networks. We continue this process until only $\lambda_{1}-\lambda_{2}$ nodes remain unaligned.

#### 2.2.3 Exposed G score

Our second proposed measure is the EGS. Here, we want to reward alignments where all the *g*-annotations are concentrated into a smaller number of towers. We imagine that *g*-annotations in a tower are aligned vertically, and the uppermost *g*-annotation in the tower is “exposed,” while the ones “below” it are not. Thus, a better alignment is one that has a smaller number of exposed GO terms. A lower EGS indicates that more *g*-annotated nodes are aligned together and fewer annotated nodes are “exposed,” resulting in a better alignment. An example of using EG can be seen in Fig. 1, where exposed G would give a score of 2 to Alignment 1 and a score of 1 to Alignment 2. For this score too, the components are lambda-sorted.

(5) $$\text{Exposed}\;G=|\{T|a_{T}\geq 1\}|$$

The calculation of EG involves the number of towers with at least one *g*-annotated node. The minimum value of EG is *λ*_1_ where all of the *g*-annotated nodes are concentrated in *λ*_1_ towers. An upper bound for the maximum value of EG is the sum of *λ_i_* values for all the networks which is the same worst-case value as in SG. The EGS is then calculated using these 2 values.

(6) $$\text{Min}(\text{Exposed}\;G)=\lambda_{1}$$

(7) $$\text{Max}(\text{Exposed}\;G)=\text{min}(n_{0},\sum_{i=1}^{k}\lambda_{i})$$

(8) $$\text{Exposed}\;G\;\text{Score}=\frac{\text{Max}(\text{Exposed}\;G)-\text{Exposed}\;G}{\text{Max}(\text{Exposed}\;G)-\text{Min}(\text{Exposed}\;G)}$$

To incorporate EGS into the final alignment score, it is subtracted from the worst-case maximum value in the numerator. This transforms this measure from a cost measure into a score between 0 and 1. So after the normalization, a higher EGS indicates a better alignment.

To better understand EGS intuitively, consider a library where each book represents a protein and is categorized by specific genres, analogous to GO terms, and these genres are organized into different sections, representing towers. Here, we want to score the organization of the books in the library. If books of a particular genre are scattered across numerous sections, finding a book becomes challenging. Conversely, if books of the same genre are concentrated in just a few sections, readers will know exactly where to go. With the same intuition, EGS rewards the alignment that concentrate each GO term into as few columns as possible and penalizes the reverse. To understand SGS, consider that in the same library analogy, when the reader is exploring a section known to contain books with their genre of interest, if there are only a few books of that genre, the reader must sift through many irrelevant options. Conversely, if the section is densely populated with books from the sought-after genre, the reader’s search becomes much more straightforward. In the same way, SGS scores the alignment based on how densely each tower contains proteins with a specific GO term.

### 2.3 Experiments

Two experiments were used to compare objective evidences against the functionality of the measures to assess their quality.

#### 2.3.1 Perfect self-alignment with controlled error rate

Our goal in this section was to produce controlled noise using self-alignment. For a species, we aligned each network with *k* instances of itself and started with a perfect self-alignment, meaning that every cluster has either all *k* copies of the same node, or is empty. Next, we swapped a specific fraction of random node pairs, choosing a network and a node randomly to reach the value of the predetermined error. Starting with a perfect self-alignment and permuting a fraction of the nodes, allowed us to know exactly what percentage of the nodes were mismatched at every step. Therefore, we were able to use this knowledge to evaluate our measures. We generated 30 alignments for each combination of *k* from 3, 5, or 7, error rates from 0 to 1, and species from human, mouse, rat, and fruit fly, resulting in a total of 3600 self-alignments. We used March 2023 BioGRID networks and the GO database for the same month and year for the first experiment (Chatr-Aryamontri *et al.* 2017). A summary of the BioGRID networks used in this article is demonstrated in the Table 1.

**Table 1.** A summary of BioGRID networks.

Nodes	Edges	Common name	Official name	Abbr
19 767	784 351	Human	H. Sapiens	HS
9165	55 797	Fruit fly	D. Melanogaster	DM
10 926	62 645	Mouse	M. Musculus	MM
3025	5773	Rat	R. Norvegicus	RN
6033	134 605	Yeast	S. Cerevisiae	SC

#### 2.3.2 Using SANA multiple network alignment on IID networks

In the next experiment, SANA network aligner was used to produce multiple network alignments (Rong *et al.* 2022). SANA is an iterative aligner driven to maximize common topology. We expected the quality of the alignments to increase with the number of iterations. We wanted to see how the measures assess the quality of alignments in different SANA iterations. IID mammalian networks were used for this experiment (Kotlyar *et al.* 2019). BioGRID networks are highly uneven in network coverage and so we cannot expect good alignments to come from topology alone (Wang *et al.* 2022b). Therefore, we specifically used IID mammalian networks because, although partly synthetic, they have a substantial (though not perfect) topological similarity by construction. To ensure meaningful results from the iterations, we selected all the GO term annotated species within the IID mammalian networks, which included human, mouse, rat, dog, and cow. Within these networks, we created 30 topology-driven alignments for each combination of *k *=* *3, and *k *=* *5 species. The summary of the information of the utilized IID mammalian networks is shown in the Table 2.

**Table 2.** A summary of IID mammalian networks.

Nodes	Common name	Official name	Abbr	TaxID
18 079	Human	H. Sapiens	HS	9606
17 529	Mouse	M. Musculus	MM	10 090
15 740	Rat	R. Norvegicus	RN	10 116
14 512	Dog	C. Familiaris	CF	9615
14 783	Cow	B. Taurus	BT	9913

The measures were also validated in comparison to the number of recovered orthologs within species. An *ortholog pair* is a pair of proteins p1 and p2, one from two different species s1 and s2, respectively, which have a common ancestor protein p in the species s which is the most recent common ancestor of s1 and s2. Thus, the proteins are expected to be highly similar in both structure and function (Fitch 1970). The definition can be extended to multiple proteins from multiple species all of whom have a common ancestor. Thus, from a functional point of view, a tower that aligns a set of orthologs all of whom have a common ancestor is “good,” and it is better the greater the number of common orthologs. Thus we can use the squared number of orthologs in a tower as a known, gold-standard surrogate for alignment quality. We looked at the midway alignments on different iterations of SANA and we counted the number of recovered orthologs, the orthologs that were correctly aligned together, in these iterations. The number of recovered orthologs provides additional objective evidence for the quality of the alignment. We then compared the trend of SGS, EGS, and the existing measures with the trend of the recovered ortholog counts.

Next, we compared existing measures and our proposed measures against the recovered ortholog fraction as a gold standard in the midway alignments on different iterations of SANA. The recovered ortholog fraction was calculated as the division of the number of recovered ortholog pairs by the total number of recovered ortholog pairs across any two species in the multiple network alignment. Recovered ortholog fraction provides an objective gold standard to measure the quality of the alignment. We compared our measures with GC, GS, and protein Semantic Similarity. GC is defined as the fraction of protein pairs with at least one shared GO annotation (Vijayan and Milenković 2018). GS is defined as the fraction of consistent towers which are the towers with at least a shared GO annotation (Gligorijević *et al.* 2016). Semantic Similarity of a tower of proteins is defined as the maximum information content of the lowest common ancestor of its proteins and it is averaged for all the aligned towers in the multiple network alignment (Kazemi and Grossglauser 2020).

Additionally, we filtered GO terms with different *λ*_1_ values and calculated SGS and EGS using only the GO terms that had a *λ*_1_ value below specified thresholds. We used this experiment to show whether the measures are successful in looking at each GO term in its own context with regard to its frequency. This experiment was done using all five IID mammalian networks.

## 3 Results

### 3.1 Perfect self-alignment with controlled error rate

**Figure 2.**
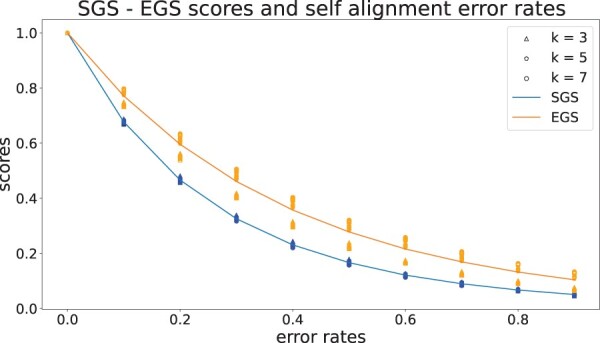
The figures show the result of the self-alignment experiment for BioGRID networks. The plot is generated using the human network and the result is similar for other species. The upper curve shows the trend of EGS and the lower curve shows the trend of SGS. The related data points are shown using same-color triangles (for *k* = 3), pentagons (for *k* = 5), and circles (for *k* = 7)

Figure 2 displays the results of the perfect self-alignment with a controlled error rate for both EGS and SGS. Each circle in the right figures represents an individual data point. As evident from the figures, the data points exhibit a very high level of proximity to each other, indicating a high degree of clustering for every value of *k* and error rate. This low variance shows that the measures consistently distinguished the quality of alignments for different states.

Additionally, both SGS and EGS measures are monotonically decreasing with the error rate. There is a strong parabolic relationship between the variables, with a quadratic model’s *R*-squared value of 0.996 for EGS and 0.983 for SGS as would be expected since our measures increase with the square of the correct (non-error) rates. The reason that higher values of *k* cause the score of the measures to decrease is that introducing the same error rate for a larger number of networks causes a higher percentage of perfect towers to be misaligned.

### 3.2 Using SANA multiple network alignment on IID networks

**Figure 3.**
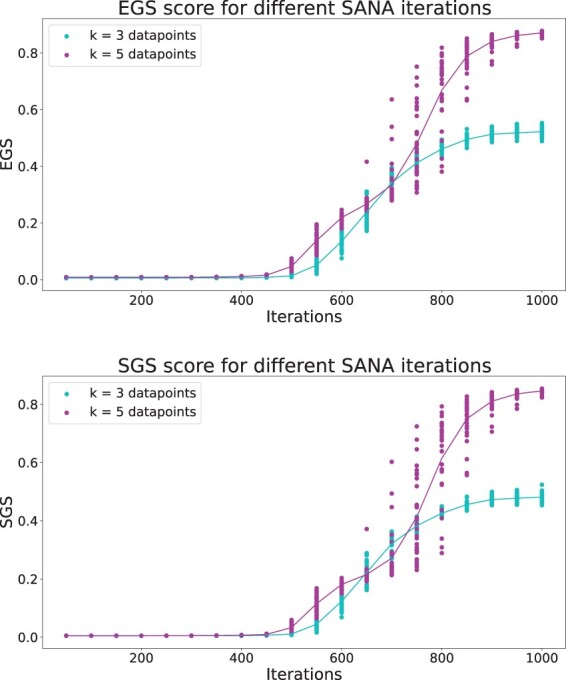
The plots show the result of exposed G and SGS, after running SANA for 1000 iterations on IID mammalian networks for 3 and 5 networks. The first figure is the result of EGS and the second one is for SGS

Figure 3 shows the result of SGS and EGS for SANA multiple network aligner on IID networks. Whereas in Fig. 2 the horizontal axis was noise level with higher noise to the right, in Fig. 3 the horizontal axis is effectively reversed, since it represents *time* during simulated annealing, with the time = 0 representing random alignments, and the alignments improving (i.e. becoming less noisy) to the right. The curves for both SGS and EGS show a monotonically increasing pattern with iterations, as illustrated in the figures. The curves for SGS and EGS produce very close absolute values, as shown in the first figure. For *k *=* *3, different sets of species were picked from the five IID mammalian networks. The randomness in the simulated annealing process of the aligner causes more variance in the quality when we are aligning 5 networks rather than 3 networks. At the final iterations on the other hand, the alignment for 3 networks has a higher quality variance which is due to having 10 different sets of network for alignment. Further analysis revealed that when the alignment happened between species with more complete networks, such as mouse, rat, and human, the alignment had better scores. This shows that more complete networks allow SANA to produce alignments that better expose functionally similar regions.

**Figure 4.**
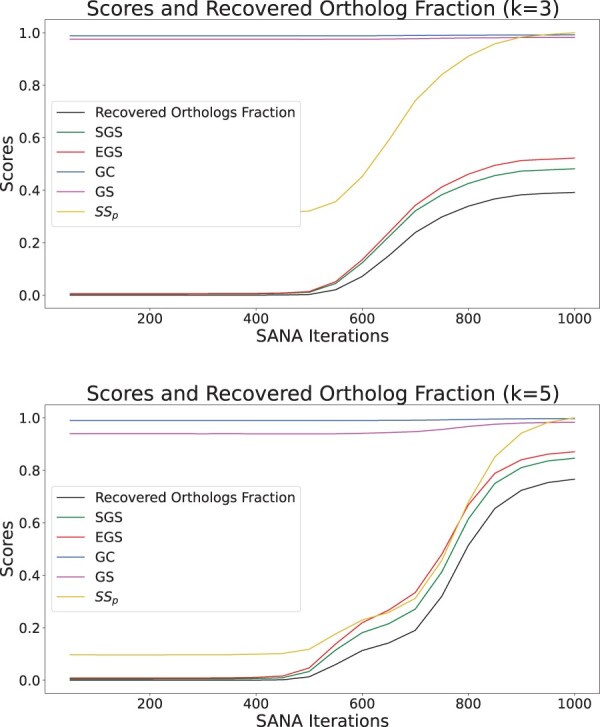
The plots show a comparison between different multiple network alignment quality measures and the fraction of recovered orthologous as the gold standard. The recovered orthologs fraction is computed in each iteration as the division of the number of recovered ortholog pairs by the total number of ortholog pairs. The first figure is the result of this experiment on three networks and the second one, on five networks. SGS, EGS, GC, GS, and protein semantic similarity (SS_p_) are compared against the recovered ortholog fraction. GC and GS are almost flat and provide no information on the quality

Figure 4 shows the result of comparing the existing measures and our proposed measures against the recovered ortholog fraction. We summarized the Spearman rank correlation, its *P*-value, and mean absolute error in Tables 3 and 4 for three and five networks. Semantic Similarity is not bounded and we used the maximum value to normalize it below one. This does not affect its Spearman rank correlation and *P*-value but enables us to calculate its mean absolute error. SGS, EGS, and recovered orthologs were also normalized only using their maximum possible values. However, the Spearman’s rank correlation showed a weak correlation with the gold standard in comparison to EGS and SGS. Although, GC and GO specificity both showed positive correlation; the correlation was less stronger than Semantic Similarity, EGS, and SGS. As explained in the introduction, GC and GS are prone to early saturation as they only check if there is a common GO term between the aligned proteins. Therefore, it is no surprise that the ratio between the score for the last iteration and the first was less than 1.05, making them useless for actual quality measurement. On the other hand, if we have a correctly aligned tower of orthologs, this would increase SS_p_ significantly as the information content of such a tower would be high. However, even if one of the orthologs are not correctly aligned and instead we have one irrelevant protein in that tower, the whole SS_p_ score for that tower would be very low. This is why although SS_p_ shows positive correlation with recovered Ortholog fraction, it has worse correlation, *P*-value, and mean absolute error and fails to fully capture the quality of the alignment.

**Table 3.** Comparison of measures with recovered ortholog fraction (*k* = 5).

	Spearman’s *ρ*	*P*-value	Mean absolute error
SGS	**0.99**	**9.5 × 10^–19^**	**0.04**
EGS	0.99	**9.5 × 10^–19^**	0.07
GC	0.75	1.4 × 10^–4^	0.78
GS	0.74	1.7 × 10^–4^	0.74
SS_p_	0.98	3.5 × 10^–15^	0.13

Bold values represent the best results for each column.

**Table 4.** Comparison of measures with recovered ortholog fraction (*k* = 3).

	Spearman’s *ρ*	*P*-value	Mean absolute error
SGS	**0.99**	**9.5 × 10^–19^**	**0.04**
EGS	0.99	**9.5 × 10^–19^**	0.07
GC	0.70	7.3 × 10^–4^	0.77
GS	0.66	1.5 × 10^–3^	0.76
SS_p_	0.95	6.2 × 10^–11^	0.42

Bold values represent the best results for each column.

Additionally, EGS and SGS had a very low mean absolute error when compared to the recovered ortholog fraction which shows they are good indicators of the quality of the alignment.

**Figure 5.**
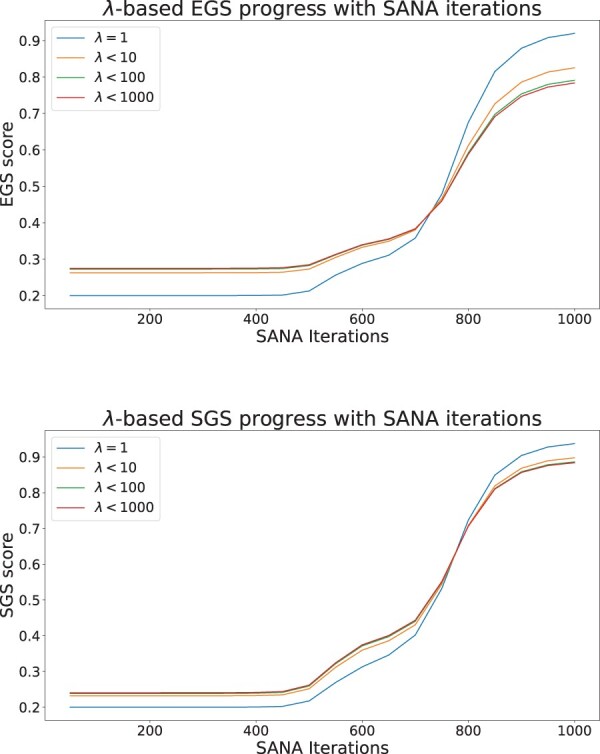
The plots show the result of using different *λ*_1_ threshold values for GO terms using 5 IID Mammalian networks. The first figure is the result for EGS and the second figure is the result for SGS. The values 1, 10, 100, and 1000 were used as threshold values. Using 10 as a threshold value means calculating the scores using only the GO terms that had a *λ*_1_ value equal to or less than 10

**Figure 6.**
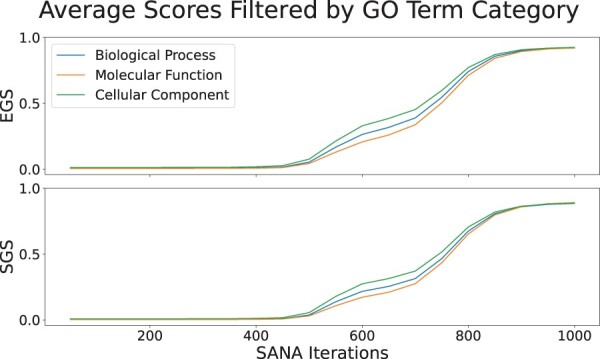
The plots demonstrate the result of only using a single category of GO terms using 5 IID Mammalian networks and SANA Iterations. The first figure is the result for EGS and the second is the result for SGS. BP, MF, and CC are represented by blue, orange, and green curve in the plots

Our measures work one GO term at a time, and the user can use any filter they want. Using frequency thresholds as demonstrated in the Fig. 5, is an example of these filters. This figure shows the result of calculating scores using a threshold value for *λ*_1_. It is important to note that we only normalized these values by their maximum since we wanted to compare the raw scores. The results show that both measures were capable of scoring the alignments using all the different threshold values. More interestingly, using 1 as a threshold for *λ*_1_ gave the best results, as it was lower than the other thresholds in the initial iterations and higher in the last ones. The reason for this is that the GO terms that have smaller *λ*_1_ are the more specific GO terms. Before running EGS and SGS, we always add the ancestors of all the GO terms to the proteins, ie if a protein is annotated by GO term, we also annotate it by its parent recursively. This ensures two things; if the proteins in the tower do not share any GO terms but the GO terms have shared ancestors, the ancestors are considered in the score. Second, we are giving a higher weight to the more specific GO terms as their ancestors would also be enforcing the score of those GO terms. For this reason, our measures perform well even when we consider different frequency threshold values. As another example of using filters, we can exclusively use the GO terms of a specific category, BP, MF, or CC to only score the alignment based on one of them. As demonstrated in the Fig. 6, all three categories have close positive correlation with SANA iterations and a small difference in their trends.

### 3.3 Functional relevance

#### 3.3.1 Scaling prediction precision of GO annotations with EGS and SGS

In this section, we show the application of EGS and SGS in annotating the missing GO terms of a species using multiple network alignment. To begin with, we adapted a method similar to the one used for GO term prediction and annotation in pairwise network alignment (Wang *et al.* 2022a). For this purpose, we created a hundred multiple network alignments using the five BioGRID networks summarized in Table 1: human, fruit fly, mouse, rat, and yeast. We used BioGRID v3.0.064, released in April 2010, as well as annotations from the GO database released the same month to predict missing protein-GO term pairs for the human network using the proteins of other species. We then used the GO database of March 2023 to validate each predicted annotation by checking whether the protein has been later annotated with the GO term by March 2023. An annotation prediction from April 2010 was considered *validated* if the annotation appeared in the GO database release of March 2023. We divided the number of validated predictions by the number of all predictions to find the precision of the predictions.

**Figure 7.**
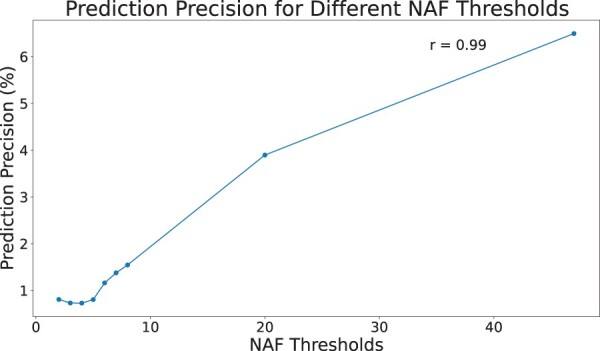
This plot shows the correlation between the prediction precision and different NAF thresholds. Each point of NAF on the plot is a threshold on NAF, meaning we only considered the pairs with NAF higher than the value as our predictions. The Pearson correlation of the prediction precision in this plot is 0.99

We extended the definition of Network Alignment Frequency from pairwise networks (Wang *et al.* 2022a) to multiple networks. We counted the occurrences of shared GO annotations in aligned triplets within each tower. The reason we considered combinations of 3 was that by considering 4 or 5 proteins at the time, we would lose valuable information. For instance, if we had a single random network in the alignment we get no predictions by requiring all 4 or 5 proteins to align together in different alignments. We first demonstrated the correlation of the prediction precision with NAF as can be viewed in Fig. 7. As expected, the prediction precision is positively correlated with NAF threshold. Additionally, it is important to note that we have results with an NAF value of 47. The chance of these results being random is $3.5\times 10^{-248}$. This number is calculated using $\left(\begin{array}{c} 99\\ 46 \end{array}\right)\times {\left(10^{-6}\right)}^{46}\times {\left({\left(\frac{999}{1000}\right)}^{2}\right)}^{63}$ as the probability of having exactly 47 occurrences of a specific triplet in 100 independently generated alignments. This is a loose upper bound on *P*-value as we are assuming only 1000 nodes in each network which in the case of human network is less than a 10th of the actual number of nodes.

**Figure 8.**
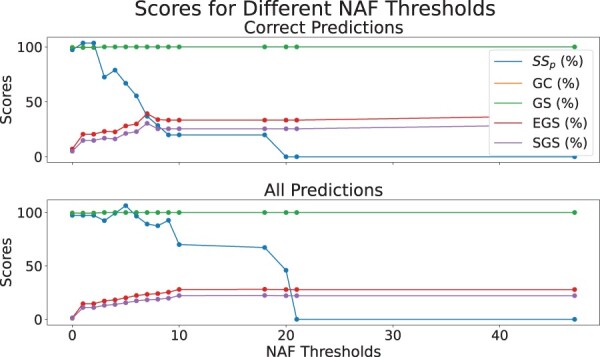
The plots show the average of different scores for all the predictions with a NAF above different thresholds. The scores shown are Protein Semantic Similarity, GC, GS, and EGS and SGS. The first plot shows the results for only the correct predictions while the second plot shows the result for all predictions. The scores are multiplied by 100

Then we plotted the average of all measures for different NAF thresholds in Fig. 8. We used each threshold to filter the protein-GO term pairs and calculated the average of the measures for the GO terms in these pairs. The measures assess the quality of the alignment of a GO term across the multiple network alignment. Therefore, while NAF provides a valuable tool in assessing the protein-GO term pair, the measures allow us to see how well the GO term has been aligned and how likely it is that they were aligned correctly in this case too. The strong correlation for EGS and SGS shows that the GO terms that are present in predictions with higher NAF thresholds have better scores as well. GC and GS are both saturated very early. Semantic Similarity has a negative correlation. Additionally, for Semantic Similarity the score for the correct predictions is lower than the average scores for all the predictions. However, for EGS (%) the score for the correct predictions is higher on average 7 points and SGS is higher on average 4 points than the all prediction EGS and SGS scores.

**Figure 9.**
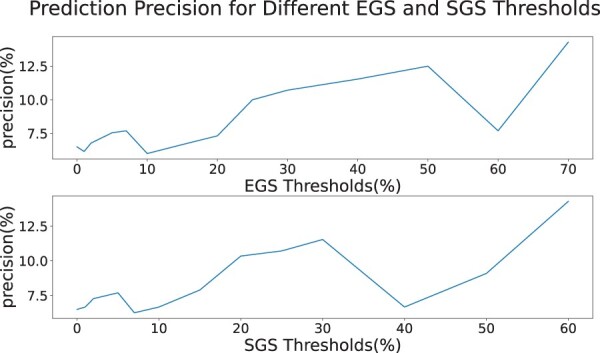
The plots show the prediction precision based on different EGS and SGS thresholds. The first plot is the precision for different EGS thresholds and the second plot is precision for different SGS thresholds

We used this idea to see how we can apply these measures to increase the precision of the predictions. For this part, we first used NAF to generate the predictions and used the pairs predicted by the highest NAF threshold, as they had the best precision and we could not reach a better precision for these networks using NAF alone. Then we filtered the predictions using different threshold values on the measures and only considered the predictions with a GO term SGS of EGS score of equal or above the threshold. The demonstrated results can be viewed in Fig. 9. This plot shows how using the measures has significantly increased the prediction precision. For both SGS and EGS, the precision has increased to more than the double of its value when we kept the GO terms with high EGS or SGS scores. The results are close in absolute values for both SGS and EGS.

## 4 Discussion

We observed a strong correlation between our scores and both alignment quality, and recovered orthologs. Additionally, we applied different thresholds on *λ*_1_ values and showed that the measures were able to produce correct results even when we use thresholds as low as $\lambda_{1}=1$. This experiment also demonstrated that even GO terms with lower frequency provided useful information about the quality of an alignment.

Lastly, we assessed the application of the measures in providing a more precise cross-species prediction of annotations. We used NAF to predict missing annotations for human proteins using April 2010 data, validated using March 2023 GO database. We demonstrated that using EGS and SGS to further filter the predictions can increase the precision from 6.5% to 14.3%. We also found a strong correlation between NAF and prediction precision, in addition to NAF and the measures, which further demonstrated the application of the measures in GO term annotation.

### 4.1 Limitations

Despite these promising results, it is essential to acknowledge an important limitation. Although each GO term is normalized using the data of its own frequency, we combined scores for different GO terms using averaging, which may have overlooked the inter-dependencies between the GO terms. A GO term *g* and its descendants, have co-occurrences in the proteins they label. Generally, *g* is a less specific GO term and we account for it when we consider the frequencies. However, in an extreme case where *g* and its descendants are all highly specific and very co-occurring, by using average in the second step, *g* and its descendant have a larger impact in the final score in comparison with other GO terms. This is not inherently bad, but if we are looking for a measure that accounts for these interdependencies, our measures should be combined with a tool that removes the extra weight based on the correlations instead of averaging.

### 4.2 Future work

In the future, we plan to further enhance our approach by computing the statistical significance of creating a multiple network alignment with a specific value for these measures at random. Additionally, the measures presented in this study have the potential to be applied to other types of multiple network alignments that are annotated with a certain ontology. Therefore, we plan to extend their usage to other types of networks such as social networks to see how they work in those settings.

## 5 Conclusion

In conclusion, our proposed measures have shown promise in assessing the quality of multiple network alignment in PPI networks. Our work has applications in predicting gene function and understanding the functional relationships between proteins. SGS offers a detailed analysis of GO terms in each tower while EGS provides a simple and effective way to evaluate the alignment as a whole. Both measures work with a single GO term while accounting for its frequency in the network.

## Data Availability

The scripts and the raw and processed data are now publicly available online.

## References

[btae476-B1] Ashburner M , Ball CA, Blake JA et al Gene ontology: tool for the unification of biology. Nat Genet 2000;25:25–9. 10802651 10.1038/75556 PMC3037419

[btae476-B2] Brückner A , Polge C, Lentze N et al Yeast two-hybrid, a powerful tool for systems biology. Int J Mol Sci 2009;10:2763–88. 19582228 10.3390/ijms10062763 PMC2705515

[btae476-B3] Chatr-Aryamontri A , Oughtred R, Boucher L et al The biogrid interaction database: 2017 update. Nucleic Acids Res 2017;45:D369–79. 27980099 10.1093/nar/gkw1102 PMC5210573

[btae476-B4] Davis D , Yaveroğlu ÖN, Malod-Dognin N et al Topology-function conservation in protein–protein interaction networks. Bioinformatics 2015;31:1632–9. 25609797 10.1093/bioinformatics/btv026 PMC4426845

[btae476-B5] Fitch WM. Distinguishing homologous from analogous proteins. Syst Biol 1970;19:99–113. 10.2307/2412448 5449325

[btae476-B6] Furuse M , Fujita K, Hiiragi T et al Claudin-1 and-2: novel integral membrane proteins localizing at tight junctions with no sequence similarity to occludin. J Cell Biol 1998;141:1539–50. 9647647 10.1083/jcb.141.7.1539 PMC2132999

[btae476-B7] Gene Ontology Consortium. The gene ontology project in 2008. Nucleic Acids Res 2008;36:D440–4. 17984083 10.1093/nar/gkm883 PMC2238979

[btae476-B8] Gillis J , Ballouz S, Pavlidis P. Bias tradeoffs in the creation and analysis of protein–protein interaction networks. J Proteomics 2014;100:44–54. 24480284 10.1016/j.jprot.2014.01.020 PMC3972268

[btae476-B9] Gligorijević V , Malod-Dognin N, Pržulj N. Fuse: multiple network alignment via data fusion. Bioinformatics 2016;32:1195–203. 26668003 10.1093/bioinformatics/btv731

[btae476-B10] Hayes WB. Exact p-values for global network alignments via combinatorial analysis of shared go terms: refango: rigorous e valuation of functional alignments of networks using gene ontology. J Math Biol 2024;88:50. 38551701 10.1007/s00285-024-02058-z PMC10980677

[btae476-B11] Kabsch W , Sander C. On the use of sequence homologies to predict protein structure: identical pentapeptides can have completely different conformations. Proc Natl Acad Sci U S A 1984;81:1075–8. 6422466 10.1073/pnas.81.4.1075 PMC344767

[btae476-B12] Kalaev M , Smoot M, Ideker T et al Networkblast: comparative analysis of protein networks. Bioinformatics 2008;24:594–6. 18174180 10.1093/bioinformatics/btm630

[btae476-B13] Kanehisa M , Goto S. KEGG: kyoto encyclopedia of genes and genomes. Nucleic Acids Res 2000;28:27–30. 10592173 10.1093/nar/28.1.27 PMC102409

[btae476-B14] Kazemi E , Grossglauser M. MPGM: scalable and accurate multiple network alignment. IEEE/ACM Trans Comput Biol Bioinform 2020;17:2040–52. 31056510 10.1109/TCBB.2019.2914050

[btae476-B15] Kimchi-Sarfaty C , Oh JM, Kim I-W et al A ‘silent’ polymorphism in the MDR1 gene changes substrate specificity. Science 2007;315:525–8. 17185560 10.1126/science.1135308

[btae476-B16] Kotlyar M , Pastrello C, Malik Z et al IID 2018 update: context-specific physical protein–protein interactions in human, model organisms and domesticated species. Nucleic Acids Res 2019;47:D581–9. 30407591 10.1093/nar/gky1037 PMC6323934

[btae476-B17] Kuchaiev O , Rašajski M, Higham DJ et al Geometric de-noising of protein-protein interaction networks. PLoS Comput Biol 2009;5:e1000454. 19662157 10.1371/journal.pcbi.1000454 PMC2711306

[btae476-B18] Milenković T , Pržulj N. Uncovering biological network function via graphlet degree signatures. Cancer Inform 2008;6:257–73. 19259413 PMC2623288

[btae476-B19] Morrone A , McCully ME, Bryan PN et al The denatured state dictates the topology of two proteins with almost identical sequence but different native structure and function. J Biol Chem 2011;286:3863–72. 21118804 10.1074/jbc.M110.155911 PMC3030387

[btae476-B20] Pennacchio LA. Insights from human/mouse genome comparisons. Mamm Genome 2003;14:429–36. 12925891 10.1007/s00335-002-4001-1

[btae476-B21] Pržulj N , Corneil DG, Jurisica I. Modeling interactome: scale-free or geometric? Bioinformatics 2004;20:3508–15. 15284103 10.1093/bioinformatics/bth436

[btae476-B22] Resnik P. Semantic similarity in a taxonomy: an information-based measure and its application to problems of ambiguity in natural language. J Artif Intell Res 1999;11:95–130.

[btae476-B23] Rong S , Wang W, Ly V et al Multi-sana: comparing measures of topological similarity for multiple network alignment. IEEE Trans Evol Computat 2022;26:1117–28.

[btae476-B24] Ruepp A , Waegele B, Lechner M et al CORUM: the comprehensive resource of mammalian protein complexes–2009. Nucleic Acids Res 2008;38:D497–501. 10.1093/nar/gkp914 PMC2808912 19884131

[btae476-B25] Schlicker A , Domingues FS, Rahnenführer J et al A new measure for functional similarity of gene products based on gene ontology. BMC Bioinformatics 2006;7:302–16. 16776819 10.1186/1471-2105-7-302 PMC1559652

[btae476-B26] Trung HT , Toan NT, Van Vinh T et al A comparative study on network alignment techniques. Expert Syst Appl 2020;140:112883.

[btae476-B27] Vijayan V , Milenković T. Multiple network alignment via multimagna++. IEEE/ACM Trans Comput Biol Bioinform 2018;15:1669–82. 28829315 10.1109/TCBB.2017.2740381

[btae476-B28] Wang S , Atkinson GR, Hayes WB. Sana: cross-species prediction of gene ontology go annotations via topological network alignment. NPJ Syst Biol Appl2022a;8:25. 35859153 10.1038/s41540-022-00232-x PMC9300714

[btae476-B29] Wang S , Chen X, Frederisy BJ et al On the current failure—but bright future—of topology-driven biological network alignment. Protein Interact Netw2022b;21:1. 10.1016/bs.apcsb.2022.05.005 35871888

[btae476-B30] Zhao N , Han JG, Shyu C-R et al Determining effects of non-synonymous snps on protein-protein interactions using supervised and semi-supervised learning. PLoS Comput Biol 2014;10:e1003592. 24784581 10.1371/journal.pcbi.1003592 PMC4006705

